# Enhanced Antimould Action of Surface Modified Copper Oxide Nanoparticles with Phenylboronic Acid Surface Functionality

**DOI:** 10.3390/biomimetics6010019

**Published:** 2021-03-15

**Authors:** Patricia Henry, Ahmed F. Halbus, Zahraa H. Athab, Vesselin N. Paunov

**Affiliations:** 1 Department of Chemistry and Biochemistry, University of Hull, Hull HU67RX, UK; patricia.sinna@gmail.com (P.H.); ahmed_fozy71@yahoo.com (A.F.H.); zahraa_athab@yahoo.com (Z.H.A.); 2 Department of Chemistry, College of Science, University of Babylon, Hilla 51001, Iraq; 3 Environmental Research Center, University of Babylon, Hilla 51001, Iraq; 4 Department of Chemistry, Nazarbayev University, Nursultan 010000, Kazakhstan

**Keywords:** antimould nanoparticles, copper oxide, 4-hydroxyphenylboronic acid, boronic acid, carbohydrates, *Aspergillus niger*, *Penicillium chrysogenum*

## Abstract

Antimould agents are widely used in different applications, such as specialty paints, building materials, wood preservation and crop protection. However, many antimould agents can be toxic to the environment. This work aims to evaluate the application of copper oxide nanoparticles (CuONPs) surface modified with boronic acid (BA) terminal groups as antimould agents. We developed CuONPs grafted with (3-glycidyloxypropyl) trimethoxysilane (GLYMO), coupled with 4-hydroxyphenylboronic acid (4-HPBA), which provided a strong boost of their action as antimould agents. We studied the antimould action of the 4-HPBA-functionalized CuONPs against two mould species: *Aspergillus niger* (*A. niger*) and *Penicillium chrysogenum* (*P. chrysogenum*). The cis-diol groups of polysaccharides expressed on the mould cell walls can form reversible covalent bonds with the BA groups attached on the CuONPs surface. This allowed them to bind strongly to the mould surface, resulting in a very substantial boost of their antimould activity, which is not based on electrostatic adhesion, as in the case of bare CuONPs. The impact of these BA-surface functionalized nanoparticles was studied by measuring the growth of the mould colonies versus time. The BA-functionalized CuONPs showed significant antimould action, compared to the untreated mould sample at the same conditions and period of time. These results can be applied for the development of more efficient antimould treatments at a lower concentration of active agent with potentially substantial economic and environmental benefits.

## 1. Introduction

Moulds belong to the fungi genre and can produce spores that propagate in the air. They grow by producing multicellular filaments (hyphae) which form an interconnected network called a “mycelium” [1]. Mould can produce secondary metabolites, or mycotoxins, which are toxic to both humans and animals [1,2]. Generally, moulds are able to grow on different surfaces, both indoors and outdoors, including buildings, food, fibers, wood and any place where moisture, organic materials and oxygen are present [2]. The growth is characterized by a non-aesthetic appearance, change of color that depends on the type of mould and the nutrients source. The growth of mould has been associated with increased adverse health effects, such as allergies, headaches, asthma and respiratory problems [3].

Some moulds, such as *Aspergillus niger*, which is one of the most common species of the genus *Aspergillus*, are able to grow on certain fruits and vegetables [4]. *A. niger* has a negative impact on grapes, apricots, onions and peanuts that causes a plant disease called “black mould” [4]. *A. niger* produces a mycotoxin called ochratoxin, which can contaminate various substrates both in the fields and after harvesting [5]. The production of mycotoxin is enhanced in the presence of suitable environmental conditions, such as damp [5,6,7,8]. Another common representative of moulds is *Penicillium chrysogenum*, which occurs in indoor environments such as in damp building materials but is also identified as a food-spoilage agent [9,10,11]. It has strains that produce moderate toxins that are highly allergenic and has also been associated with asthma [12,13]. Various antimould agents have been added by manufacturers to building products to prevent mould growth. Quaternary ammonium salts, copper salts and other types of antimould agents were used in many applications, such as mould-resistant paints and wood preservatives. However, these antimould agents show certain level of toxicity to humans and can also be harmful to the environment [14,15]. Therefore, there is a great need to develop more efficient antimould agents and formulations that can be applied at lower concentrations that are suitable for indoor and outdoor applications.

We envisage that this can be achieved by using an innovative approach based on functionalized CuO nanoparticles (CuONPs) [16,17], which have been originally developed as antibacterial agents. It was found that they bind very strongly to their cell walls, due to the abundance of cis-diol groups on the surface of both Gram-positive and Gram-negative bacteria. In the present work, we further explore their ability to target mould. Halbus et al. [18,19] found that phenylboronic acid-functionalized CuONPs and rough silica NPs can also be effective as anti-yeast agents. Similar effects and functionality have been discussed elsewhere for a range of other surface-modified inorganic nanoparticles, e.g., TiO_2_NPs [20,21], ZnONPs [22] and Mg(OH)_2_NPs [23], which proved to be very efficient antibacterial, antialgal and antifungal agents that can reduce the applied amount, lower the overall toxicity of the fungicide formulation and potentially decrease its environmental impact. Other promising antimicrobial strategies involve using nanocarrier-loaded antimicrobial agents with cationic coating to enhance their particle–cell adhesion and accumulation at microbial cell walls [24,25,26]. Some of these cationic nanoparticle formulations rely on electrostatic attraction with the negatively charged cell walls of microbial cells. Recently, active nanocarriers of both antibiotic and antifungal agents were developed with protease coating that can digest their way through biofilms delivering their payload to the embedded microbial cells [27,28,29]. However, in antimould applications, the strategy of using solely such electrostatic binding to the mould can be challenging due to the presence of other anionic species in the hyphae immediate environment which can render the treatment ineffective. Here we probe the antimould effect of engineered CuONPs with boronic acid surface functionality to design a non-electrostatic mechanism for their attachment to the mould hyphae. This is expected to amplify their accumulation on the mould cell walls despite the presence of other anionic moieties in the local environment. Our idea here is to graft boronic acid (BA) surface groups on the CuONPs, which can covalently bind to various glycoproteins and carbohydrates that are abundant on the mould hyphae walls. We used *A. niger* and *P. chrysogenum* as model mould species, to examine the antimould activity of the 4-HPBA functionalized CuONPs, compared with non-modified (bare) CuONPs. We also experimented with the mode of delivery of the nanoparticles to the mould and its culture medium, which greatly impacts on their antimould effect. We envisage that the way the nanoparticles treatment is delivered to the mould sample is important for their effectiveness as antimould agents, since the hyphae and the spores can be compartmentalized in different parts of the colonized substrate.

## 2. Materials and Methods

### 2.1. Materials

Deionized water was used in all the experiments, obtained from the reverse osmosis Milli-Q filtration station after the resistivity of the water reached 18 MΩ cm^−1^ (at 25 °C). Sodium hydroxide, NaOH, and hydrochloric acid, HCl (both 99.6%), were sourced from Fisher Scientific, UK. CuCl_2_ (99%, Sigma Aldrich, Gillingham, UK) was used as a precursor for the synthesis of CuONPs. Then, 4-hydroxyphenylboronic acid (4-HPBA), (3-glycidyloxypropyl) trimethoxysilane (GLYMO) and the culture medium of the mould (potato dextrose agar (PDA)) and ethanol (>99.97%) were purchased from Sigma-Aldrich, Gillingham, UK. Non-impregnated 5 mm filter paper discs, as well as the two mould species examined in this research, *A. niger* and *P. chrysogenum*, were purchased from Blades Biological, Kent, UK (a local UK distributor of www.carolina.com). The glassware used in all experiments was cleaned, using deionized water and commercial detergent, and then with absolute ethanol and rinsed with deionized water.

Glassware was oven-dried at 65 °C. Dettol Antibacterial Mould and Mildew Remover^TM^ spray was purchased from a local Tesco store, and ethanol spray was used to clean the bench, to minimize the risk of spreading of mould spores and cross-contamination.

### 2.2. Preparation of the Mould Growth Medium

First, 40 g of potato dextrose agar (PDA) was dispersed in 1000 mL of deionized water. Then the solution was boiled until the PDA was dissolved. The agar was autoclaved at 121 °C, for 15 min, and the flask was cooled for a further 15 min. Then the cover of the Petri dish was left open at just enough of an angle to pour in the medium. The agar solution was poured slowly, to avoid formation of bubbles. A Bunsen burner flame was passed over the surface of the agar several times (Supplementary Materials Figure S1), to ensure aseptic conditions. Enough PDA solution was poured to fill half the Petri dish, and the dishes were left undisturbed until the agar solution gelled at 25 °C.

### 2.3. Seeding of the Mould Samples into the PDA Loaded Petri Dishes

The hood was cleaned prior to use by spraying with ethanol before the preparation of the Petri dishes with the PDA. The flame of a Bunsen burner was used to sterilize the mouth of the tubes before and after use. Using a sterile micropipette, we transferred one drop to the center of a Petri dish. The lid of the Petri dish was closed and labeled with the name of the culture and the date; then a thin strip of parafilm was wrapped around the sides of the plate, to cover the opening. The Petri dishes were placed in an incubator, at 25 °C. After 6 days, a small amount of the grown mould was collected and dispersed in a sample of autoclaved water.

### 2.4. Assessment of the Antimould Activity of Surface Functionalized CuONPs towards A. niger and P. chrysogenum

**Figure 1:**
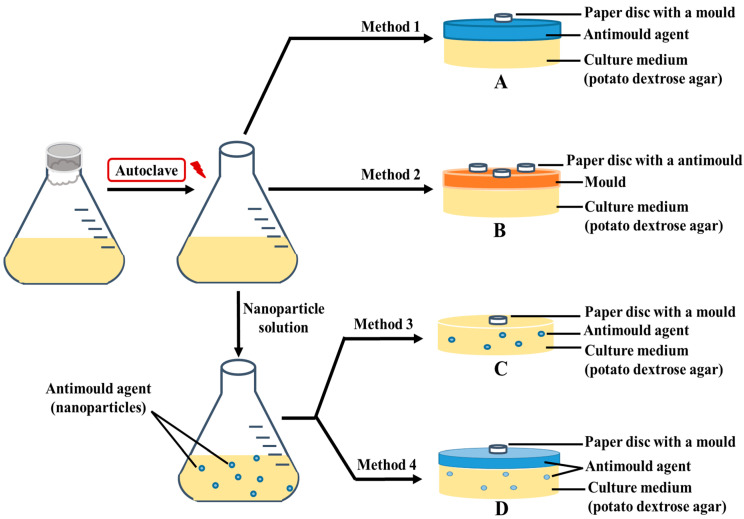
Schematics of the four different settings of application of the functionalized antimould CuONPs nanoparticles to the mould species explored in this study: (**A**) Method 1 involved spreading liquid antimould formulation on a potato dextrose agar (PDA)–gel plate and placing a paper disc impregnated with a mould suspension in the center. (**B**) Method 2 involved spreading the mould suspension over the whole PDA–gel plate surface and then placing a paper disc impregnated with antimould agent in the center. (**C**) Method 3 involved mixing of the liquid PDA-media with the antimould formulation, gelling it and placing a paper disc impregnated with a mould suspension in the center. (**D**) Method 4 was a combination of Method 1 and Method 3 and involved mixing the liquid PDA with the antimould formulation, gelling it and then spreading a sample of liquid antimould formulation on top of the PDA–gel plate. The method also included placing a paper disc impregnated with a mould suspension in the center of the gel plate. The PDA gel plates were then placed vertically, to eliminate any excess liquid on the gel surface.

First, 4-HPBA and GLYMO functionalized CuONPs labeled as CuONPs/GLYMO and CuONPs/GLYMO/4-HPBA were prepared as described elsewhere [17]. There was a specific procedure followed for preparation of Petri dishes with different medium, with small differences in the protocol as described below. PDA is the recommended growth media for these types of mould. In the first method, Petri dishes loaded with pre-prepared PDA media were used to grow the mould samples and test the antimould activity of the CuONPs formulations; 0.5 mL of each CuONPs suspension sample was put on the surface of the PDA-loaded Petri; the dish was tilted to disperse the antimould formulation evenly, and the PDA–gel plates were left for one hour to adsorb the solution. Non-impregnated 5 mm discs were immersed into the mould solution tube, using tweezers, taking care to remove excess mould solution on the disc (Figure 1A). When the disc was just damp, it was placed in the middle of the PDA-loaded Petri dish, which was then sealed with parafilm M tape. The samples were incubated at 25 °C, and the stage of the colony growth in each sample was monitored by photographing the PDA–gel plate on different days. The ImageJ program and ruler were used to calculate the growth diameter of the mould on the PDA–gel plate. In the second method, this was done in reverse, in order to assess if this protocol was better or not (Figure 1B). In the third method, the protocol consisted of mixing the liquid PDA liquid media with the antimould formulation before being gelled in the Petri dish (Figure 1C). We had to follow a specific protocol, because the antimould formulation had to work when we mixed it with the PDA solution. It apparently proved to be the one of the most successful ways to kill and suppress all moulds in the sample. In the fourth method, we used a combination of the first and the third method. Thus, to strengthen the antimould action, we mixed the antimould formulation with PDA liquid solution, and then a fixed amount of the liquid antimould formulation was added on the top of the mixed and gelled PDA medium (Figure 1D). Below, we discuss the efficiency of antimould formulations of bare and surface functionalized CuONPs applied to mould samples of the two different species, using all four methods of their delivery.

## 3. Results and Discussion

### 3.1. The Antimould Activity of Nanoparticle with A. niger and P. chrysogenum

#### 3.1.1. Method 1—Antimould Agent Applied on the PDA–Gel Plate Surface

**Figure 2:**
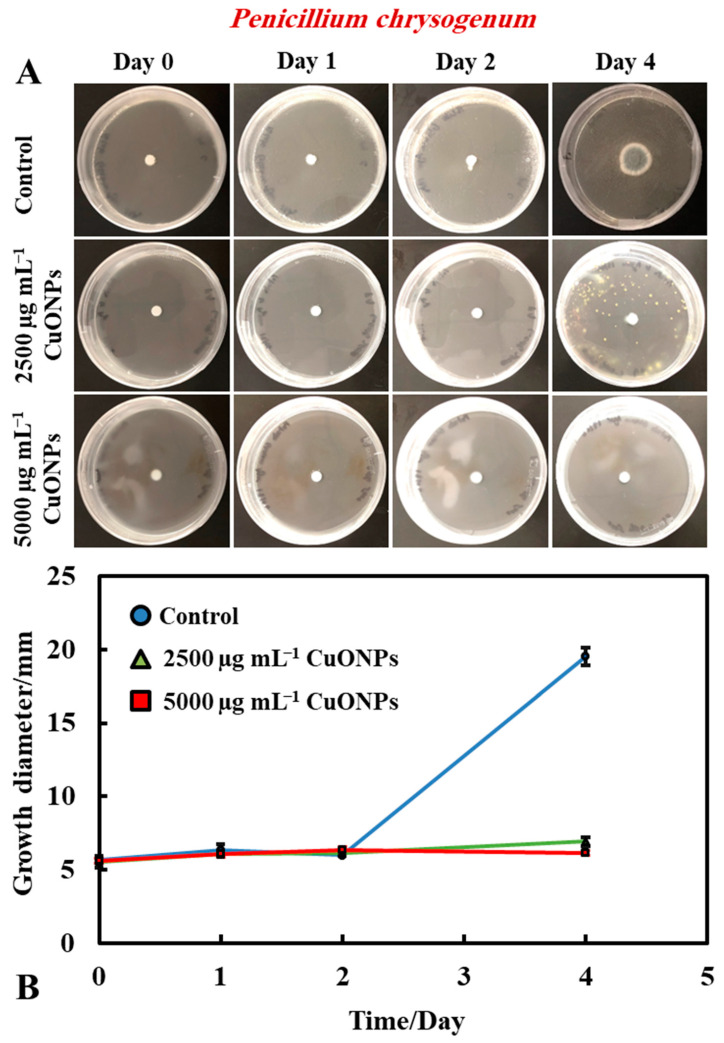
(**A**) Digital photographs of the Petri dishes containing *P. chrysogenum* seeded in the center and treated with an antimould dispersion at 2500 and 5000 µg mL^−1^ CuONPs on the top of the medium, grown for four days, at 25 °C. (**B**) Growth diameter of a circular spot of *P. chrysogenum* culture versus time incubated at 25 °C on a PDA medium for different CuONPs concentrations. The solid lines are guides to the eye.

In order to measure and determine the antimould action of the tested functionalized CuONPs antimould suspensions, a control sample for each type of mould (*A. niger* and *P. chrysogenum*) was grown for comparison with the tested formula that was prepared and characterized (Figure 2A,B). Petri dishes were prepared, using PDA as a culture medium for mould growth. A 5 mm paper disc was placed inside a solution of the chosen mould suspension and then put on the PDA–gel plate. The mould growth rate was characterized by measuring the growth diameter of the mould colony (mm) as a function of time (days). Figure 2A,B shows that the mould in the control sample grows faster than other Petri dishes treated with the CuONPs antimould formulations. Typically, the mould sample grew until it reached ~20 mm in diameter after day four, while mould samples treated with the CuONPs-based antimould formulations did not grow at all, and the sample diameter stayed at 5 mm.

**Figure 3:**
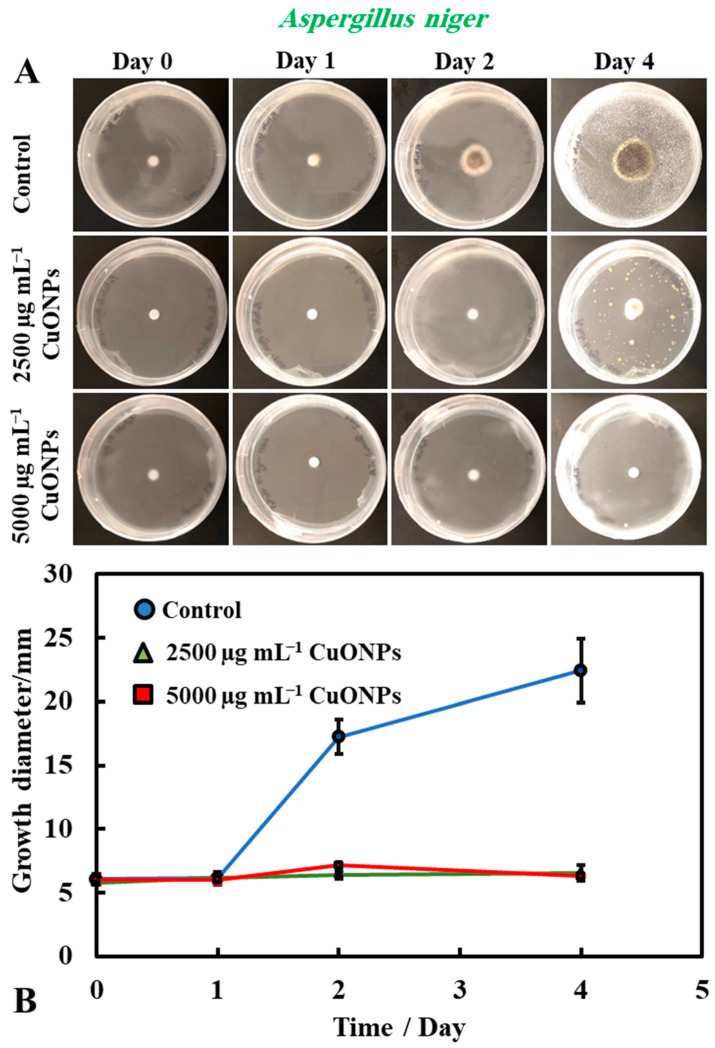
(**A**) Digital photographs of the PDA–gel plates containing *A. niger* seeded in the center and with bare CuONPs as an antimould agent at testing concentration of 2500 and 5000 µg mL^−1^, respectively, applied on the top of the PDA medium, and monitored over a period of four days, in an incubator, at 25 °C. (**B**) Growth diameter of a circular spot of *A. niger* seeded on paper disc is presented as a function of time, comparing the antimould effect of the CuONPs. The solid lines are guides to the eye.

It was found that bare CuONPs on their own had a significant antimould activity against *P. chrysogenum* at concentrations of 2500 and 5000 µg mL^−1^ and suppressed the mould growth. The same experiment was repeated with *A. niger*, to check if the bare CuONPs are also a good antimould agent across different mould species (Figure 3A,B). Here, we found a similar trend to the experiment with *P. chrysogenum.* The control sample of this mould was kept at the same conditions without treatment with the antimould agent for the same period. In controls, the mould colony grew after day two to 16 mm in diameter. It was found that the growth rate of *A. niger* decreased significantly, compared with the control (Figure 3B). The data in Figure 3 show that the growth spot vs. time did not change significantly from days two to four, apart from the control. All tested CuONPs concentrations used seem effective in subduing the mould growth. The bare CuONPs were found to be effective antimould agent, as *A. niger* did not spread on the PDA plates at CuONPs concentrations of 2500 and 5000 µg mL^−1^, respectively, and the growth diameter stayed around 5 mm. The growth rate of both *P. chrysogenum* and *A. niger* also decreased with the increase of the antimould-agent concentration. This effect can be explained with the positive surface charge of the bare CuONPs, which are cationic particles at neutral pH [17] (see Supplementary Materials Figure S2), and their strong electrostatic attraction to the anionic surface of the mould hyphae. It is likely that, due to their surface roughness, the adhered CuONPs fracture the cell membranes, as recently established for several bacterial species, as well as yeast [17,18,19,22,23].

#### 3.1.2. Method 2—Antimould Agent on Paper Disc with Mould Seeded on PDA-Plate

**Figure 4:**
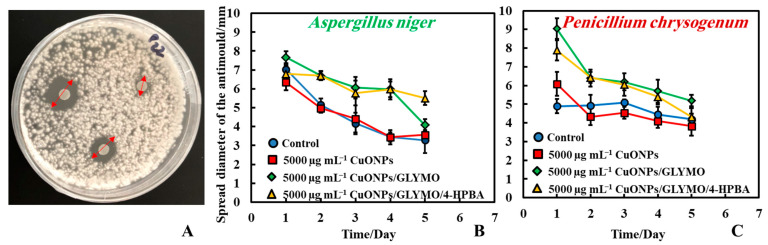
(**A**) Digital photograph of the PDA–gel plate for Method 2, showing the measured size of the mould growth inhibition zone around the antimould agent sample. Here, the paper discs were impregnated with the antimould agent, while the whole gel plate was seeded with a mould suspension. Graphs of the growth inhibition zone diameter of the antimould with (**B**) *A. niger* and (**C**) *P. chrysogenum*, over the whole PDA–gel plate as a function of time. The solid lines are guides to the eye.

The antimould activity of the CuONPs surface-functionalized with GLYMO and 4-HPBA was also tested against both moulds (*P. chrysogenum* and *A. niger*) at 5000 µg mL^−1^ concentration of the antimould agent. In this method, we deposited three paper discs impregnated with the antimould agent (Figure 4A). Here, Method 2 was used (see Figure 1) where the seeding with mould spores was done over all the surface of the PDA–gel plate initially, while the filter paper disks were impregnated with the antimould NPs formulation. The obtained trend is just the opposite to the results presented on Figure 3 where Method 1 was used. Therefore, the antimould effect is proportional to the diameter of the void space around the disk. One can see that the NPs treated filter paper disks show very large voids around which corresponds to more effective antimould action for CuONPs/GLYMO/4-HPBA. Note that a space void of mould growth developed around the paper disc, showing that Method 2 was also a good way to conduct the testing. It was found that the surface-functionalized nanoparticles (CuONPs/GLYMO/4-HPBA) displayed a strong antimould activity against both *P. chrysogenum* and *A. niger* (Figure 4B,C).

The controls sample showed that the mould spread around the paper disc and encroached it with the visible diameter of the disc being only ~3.5 mm. CuONPs/GLYMO/4-HPBA surface functionalized nanoparticles at 5000 µg mL^−1^ were more efficient (Figure 4B); the visible part of the disc stayed around 5.5 mm in diameter and resisted the mould growth. The same experiment with *P. chrysogenum* showed consistently similar results (Figure 4C). At a concentration of 5000 µg mL^−1^ CuONPs/GLYMO/4-HPBA, the mould depletion zone around the disc was around 5.5 mm, while the disc in the control Petri dish was around 4.5 mm. One can see that for the surface functionalized CuONPs the spread diameter was 8–9 mm on day one and reached 4–6 mm in day five. The control kept the same diameter up to day three and decreased in day five.

#### 3.1.3. Method 3—A Mixture of Antimould Formulation with the Growth Medium

We tested the antimould properties of the functionalized nanoparticles CuONPs/GLYMO/4-HPBA against *P. chrysogenum* and *A. niger* by mixing them with the PDA medium before the gel sets on the plate. In this method, we explored the effect of the nanoparticles when they are dispersed within the PDA–gel matrix. The rationale behind this is that the hyphae of both types of mould grow through the PDA–gel and propagate, leaving the possibility for the hyphae to bypass any antimould nanoparticle formulation when it is applied only on the surface of the PDA–gel plate. Since surface application of the CuONPs suspension may lead to an ineffective penetration through the bulk of the gel, we explored this effect systematically, in order to compare with the results from Method 1.

**Figure 5:**
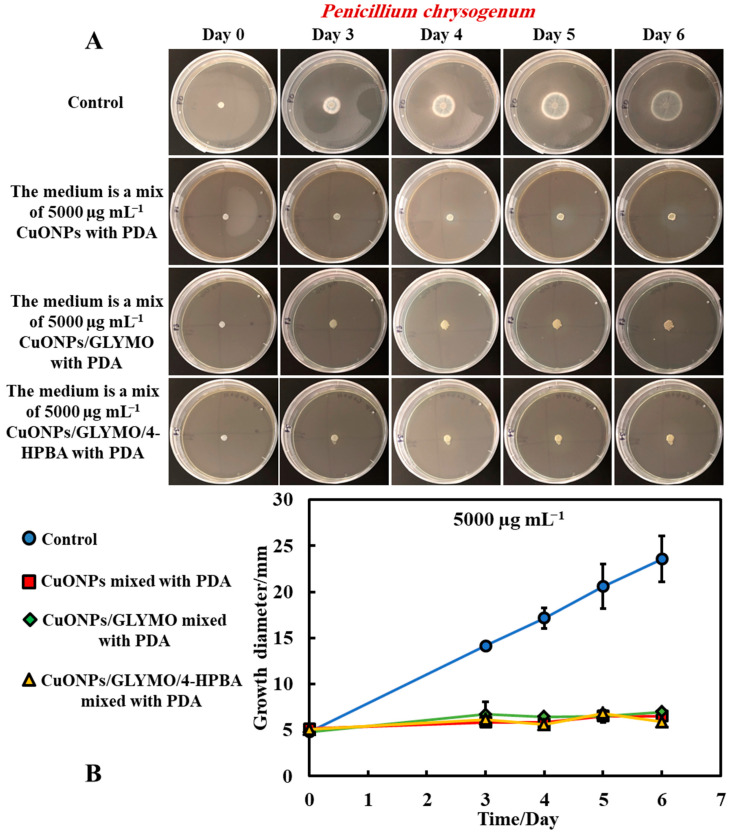
(**A**) Digital photographs of the Petri dishes with PDA medium containing *P. chrysogenum* in the center and with antimould agent at concentration of 5000 µg mL^−1^ CuONPs (bare or functionalized) mixed with the PDA growth medium and incubated for six days, at 25 °C. (**B**) Growth of a circular spot of seeded *P. chrysogenum* as a function of time for three different types of antimould nanoparticles: bare CuONPs, CuONPs/GLYMO and CuONPs/GLYMO/4-HPBA applied to the medium, according to Method 3. The solid lines are guides to the eye.

**Figure 6:**
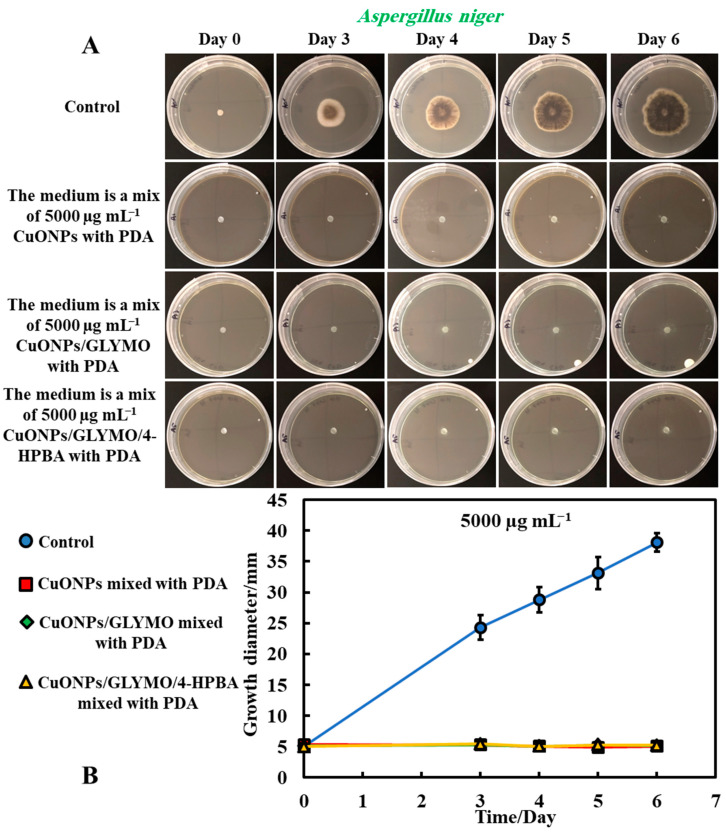
(**A**) Digital photographs of the Petri dishes with PDA medium containing *A. niger* in the center and with antimould agent at concentration of 5000 µg mL^−1^ CuONPs (bare or functionalized) inside the medium, incubated for six days, at 25 °C. (**B**) Growth of a circular spot of seeded *A. niger* as a function of time for three different types of antimould nanoparticles: bare CuONPs, CuONPs/GLYMO and CuONPs/GLYMO/4-HPBA applied to the PDA medium according to Method 3. The solid lines are guides to the eye.

Figure 5 and Figure 6 present the results of the antimould assays of both moulds species where the control samples of untreated mould were compared with the ones treated with bare CuONPs, CuONPs/GLYMO and CuONPs/GLYMO/4-HPBA. Note that, when dispersed in the gel matrix, the cationic bare CuONPs were also showing an antimould impact on *P. chrysogenum* and *A. niger*. We found that a 5000 µg mL^−1^ concentration of bare CuONPs, CuONPs/GLYMO and CuONPs/GLYMO/4-HPBA reduced the *P. chrysogenum* and *A. niger* growth rate several-fold, as compared with the control samples of the untreated mould (Figure 5A,B and Figure 6A,B). A strong impact of the bare CuONPs on *P. chrysogenum* and *A. niger* was observed after six days of incubation at 25 °C. The positive surface charge of the bare CuONPs (Supplementary Materials Figure S3) is considered to be an important factor for the interactions with the mould cell membranes, which contribute to their accumulation on the hyphae and spores surface and manifests itself into high antimould activity.

Figure 5B and Figure 6B show that, without an antimould agent, the mould has a growth diameter of 24 mm for *P chrysogenum* and 40 mm for *A. niger* after six days, while in the presence of the antimould agent, the mould did not grow, with the growth diameter staying at 5 mm (the size of the paper disc). It shows that, when dispersed throughout the matrix of the growth media, antimould nanoparticles effectively kill the spreading mould and suppress its growth. Note that CuONP_S_/GLYMO is an anionic nanoparticle (Supplementary Materials Figure S4, ESI), i.e., it has a negative surface charge at neutral pH and therefore lacks electrostatic adhesion to the negatively charged cell walls. However, they show a similar antimould effect to the bare CuONPs, which are cationic particles. One possible explanation could be that, at this concentration (5000 μg mL^−1^), the medium is so saturated with these nanoparticles that the growth of the hyphae in the bulk of the medium could potentially lead to a close contact of their cell walls with the trapped nanoparticles, thus having an adverse effect on the mould.

The introduction of a secondary functionalization of these anionic nanoparticles by conjugation of the GLYMO groups with 4-HPBA also gives anionic nanoparticles (CuONPs/GLYMO/4-HPBA)—see Supplementary Materials Figure S4. However, despite their negative surface charge, CuONPs/GLYMO/4-HPBA are also showing strong antimould impact as it can be seen in Figure 5B and Figure 6B. These results call for some discussion about the possible factors that may contribute to the enhanced antimould action of the CuONPs/GLYMO/4-HPBA. It has been shown previously in antibacterial studies [17,18] that ligands with BA-functionality can covalently bind with compounds having cis-diol groups, like glycated-proteins, saccharides and nucleotides [17,30,31,32,33]. We envisage that, despite their negative surface charge, the anionic nanoparticles CuONPs/GLYMO/4-HPBA are showing significant antimould impact on both types of mould due to their covalent binding to the mould cell-wall constituents.

Mould hyphae cells are surrounded by an outer cell membrane containing lipopolysaccharides (LPSs) with many cis-diol groups [17,18,31,34,35,36,37]. The strong (covalent) interactions between the cis-diol groups from the LPS-layer and the boronic acid terminal group of the CuONPs/GLYMO/4-HPBA particles led to the particle build-up on the mould cell membranes. In contrast, the adhesion of the bare CuONPs to the *A. niger* and *P. chrysogenum* membrane is largely driven by electrostatic interactions, whereas the CuONPs/GLYMO/4-HPBA binds to the surface-expressed saccharides through the formation of boronic esters. This facilitates their accumulation on the hyphae and makes them much more effective against *P. chrysogenum* and *A. niger*. Comparing the effects of the three different types of CuONPs (bare and functionalized) shows, surprisingly, the same strong effect on moulds that can be explained by the high nanoparticle concentration that saturates both surface and the bulk of the PDA growth media.

**Figure 7:**
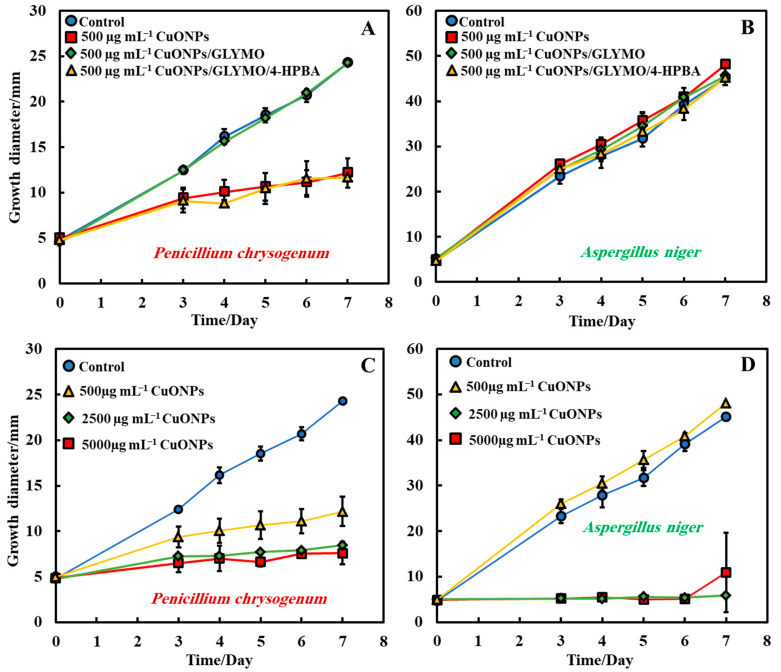
Growth diameter of a circular spot of (**A**) *P. chrysogenum* and (**B**) *A. niger* as a function of time to compare the antimould impact of the nanoparticles applied by using Method 3. Comparison of the growth diameter of a circular spot of (**C**) *P. chrysogenum* and (**D**) *A. niger* at different concentrations of the bare CuONPs. The solid lines are guides to the eye.

The antimould activity of the surface-functionalized CuONPs with GLYMO and 4-HPBA was also tested against both moulds, *P. chrysogenum* and *A. niger*, at 500 µg mL^−1^ nanoparticle concentration (Figure 7A,B). Each antimould suspension was tested separately on each type of mould according to Method 3. It was found that both CuONPs and CuONPs/GLYMO/4-HPBA have a high antimould activity against *P. chrysogenum* (Figure 7A) at a concentration of 500 µg mL^−1^ but showed no substantial antimould activity against *Aspergillus niger*. This can be seen in Figure 7B, which represents the growth of mould on day seven of 500 µg mL^−1^ antimould agent tested against *A. niger*. Figure 7C,D shows the diameter growth of *P. chrysogenum* and *A. niger* versus time for different concentrations of CuONPs. Figure 7C shows that the antimould activity of the CuONPs has an effect at 500 µg mL^−1^ with the *P. chrysogenum*, with the mould spot growing to a diameter of 12 mm at 500 µg mL^−1^ (yellow line), while the *P. chrysogenum* spot grew to a diameter of 6 mm at 2500 and 5000 µg mL^−1^ of the same antimould agent. Note that the cationic bare CuONPs show a very significant antimould effect on *P. chrysogenum* even at lower nanoparticle concentrations. Our working hypothesis is that the strong antimould action can be explained by the direct attraction of the cationic CuONPs with the anionic cell walls of *A. niger* (Supplementary Materials Figure S5) and *P. chrysogenum* (Supplementary Materials Figure S6) cell walls. On the other hand, the mould growth inhibition was different between the three concentrations of CuONPs with *A. niger*. Figure 7D shows that higher concentrations (2500 and 5000 µg mL^−1^) showed greater antimould effect compared with the control (blue line) and 500 µg mL^−1^ CuONPs (yellow line). The differences between the CuONPs impact on *A. niger* and *P. chrysogenum* can be potentially explained with the thicker cell wall of *A. niger*, compared with *P. chrysogenum*; the data suggest that it takes a much higher antimould nanoparticle concentration to impact the *A. niger* growth rate than *P. chrysogenum*. This cannot be explained with electrostatic interactions, as both moulds have a similar surface charge. Note that zeta-potential measurements of the spores of *A. niger* and *P. chrysogenum* (see Supplementary Materials Figures S5 and S6) indicate that the average zeta-potentials are both negative, −27 mV and −23 mV, respectively.

#### 3.1.4. Method 4—Antimould Agent in the Bulk and the Surface of the Growth Media

**Figure 8:**
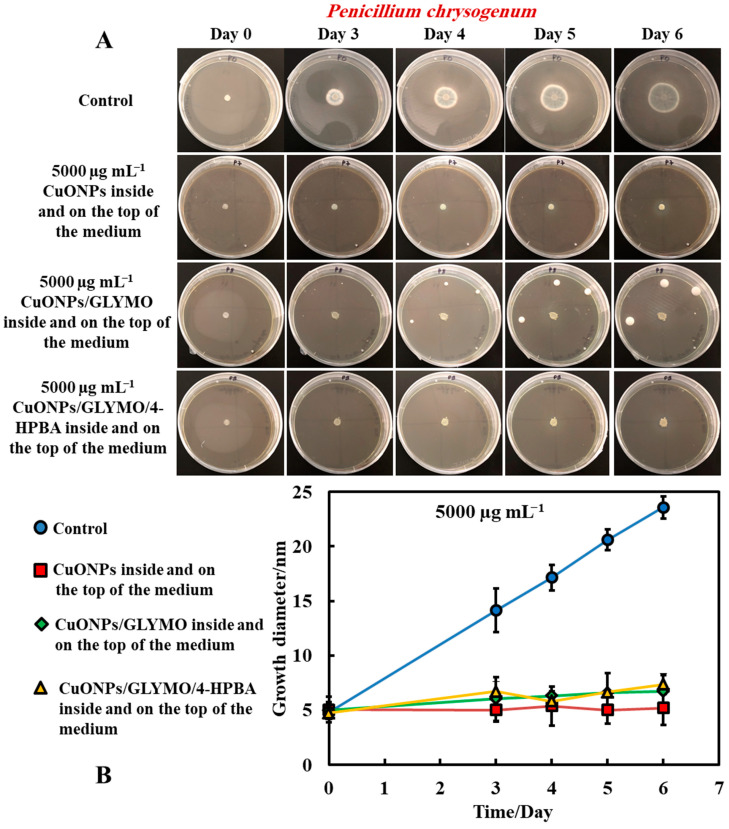
(**A**) Digital photographs of the PGA–gel plates containing *P. chrysogenum* in the center (impregnated paper disc) and with antimould agent at 5000 µg mL^−1^ inside and on the top of the medium (Method 4), monitored for six days, in an incubator, at 25 °C. (**B**) Growth diameter of a circular spot of *P. chrysogenum* as a function of time for bare and surface-functionalized CuONPs. The solid lines are guides to the eye.

**Figure 9:**
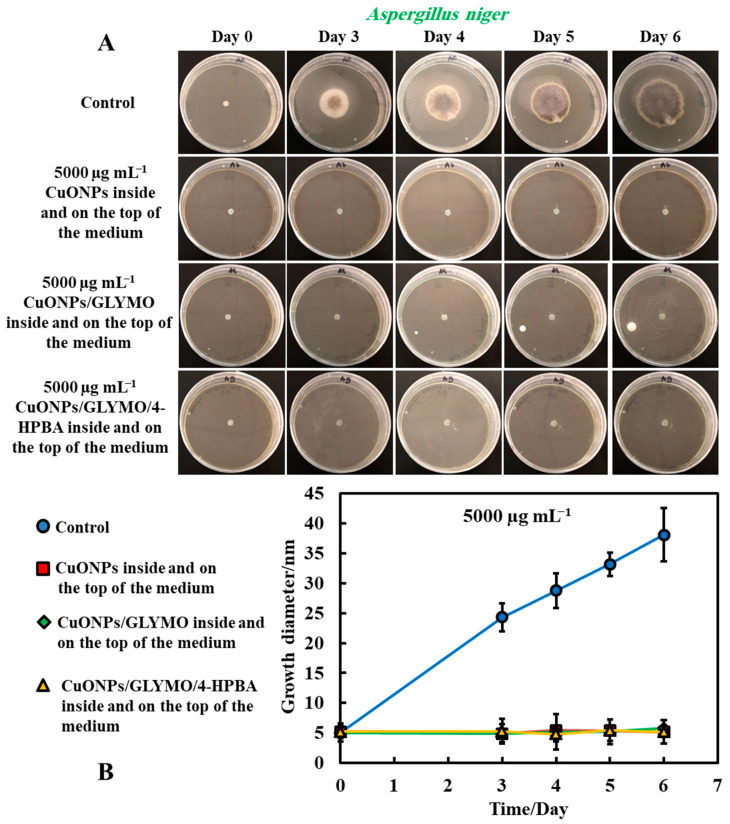
(**A**) Digital photographs of the PDA–gel plates containing *A. niger* in the center (impregnated paper disc) and with antimould agent at 5000 µg mL^−1^ inside and on the top of the medium (Method 4), monitored for six days, in an incubator, at 25 °C. (**B**) Growth diameter of a circular spot of *A. niger* as a function of time for bare and surface-functionalized CuONPs. The solid lines are guides to the eye.

The same procedure used in Method 3 was followed as described above. Method 3 worked very well with mixing of the antimould agent (CuONPs suspensions) with the PDA media. To further strengthen the antimould action, we mixed the antimould suspension with the PDA media while hot and in liquid state, and after its gelling, we also added a liquid antimould agent on the top of the gelled medium, as described in Method 1. In this way, we had the antimould agent both in the bulk of the growth media and on its surface. Figure 8 and Figure 9 demonstrate the antimould impact of the suspensions of bare CuONPs, CuONPs/GLYMO and CuONPs/GLYMO/4-HPBA at different particle concentrations on *P. chrysogenum* and *A. niger* at different times. The green line and symbols in Figure 8 indicate that the GLYMO-functionalized CuONPs are as effective as the 4-HPBA/GLYMO functionalized CuONPs due to the high overall concentration. Control samples of *P. chrysogenum* and *A. niger* were kept at the same conditions without treatment with antimould agent for the same period. The data in Figure 8 and Figure 9 indicate that the bare and surface functionalized CuONPs have an extremely strong impact on *P. chrysogenum* and *A. niger*. The growth rate of the *P. chrysogenum* decreased after day six. As it can clearly be seen, on day six, the growth diameter was around 5 mm, compared with the control samples of diameter 24 mm (Figure 8B). The explanation is that, in Methods 3 and 4, where the antimould formulation is applied in the bulk of the PDA–gel, the hyphae of both types of mould cannot grow within, and this has a strong effect on their propagation—the latter was well suppressed for all formulations, over four days.

These findings were like the ones with *A. niger* at high concentrations of the antimould agent which can also be attributed to the attraction of the surface rough cationic particles with the negatively charged mould membrane, potentially leading to their local fracturing, as described by others for bacteria [17], microalgae and yeast [18]. The data in Figure 9B show that the growth diameter of the *A. niger* spot in the presence of with antimould agent was found to be about 5 mm, compared with the untreated sample of 40 mm. These results demonstrate that the CuONPs have a strong impact on mould. This also could be explained by the cell walls of *P. chrysogenum* and *A. niger*, which are both negatively charged, whereas the bare CuONPs are positively charged in aqueous dispersion (below their isoelectric point at pH 9) (Supplementary Materials Figure S3). Therefore, the bare CuONPs were able to electrostatically adhere on the negatively charged hyphae surface, leading to the damage of their cell membrane at sufficiently high nanoparticle concentrations.

Another possible explanation for this result is that the rough surface of the CuONPs/GLYMO/4-HPBA nanoparticles [17] forces the cell membrane of the *P. chrysogenum* and *A. niger* to follow closely the particle topology because of formation of strong covalent bonds between the 4-HPBA terminal groups on the CuONPs surface and the cis-diol groups on the mould cell membrane, leading to membrane dislocation and the killing the mould cells.

## 4. Conclusions

The antimould action of bare- and surface-functionalized copper oxide nanoparticles was explored on two different types of mould, *P. chrysogenum* and *A. niger*. We demonstrated that, by surface grafting GLYMO and 4-HPBA on CuONPs, one can produce formulations which are substantially more effective against *P. chrysogenum* and *A. niger*, compared to untreated sample (control sample) at the same conditions. The 4-HPBA terminal coating produced a surface functionality that allows the CuONPs/GLYMO/4-HPBA to reversibly form covalent bonds with the cis-diol groups from carbohydrates and glycoproteins expressed on the mould hyphae cell walls of both *P. chrysogenum* and *A. niger*. Our results showed that both bare- and surface-functionalized CuONPs tested prevent the growth of both types of mould at high particle concentrations (5000 μg mL^−1^). The setting of experiment that showed the best result was Method 4: formulating the nanoparticles treatment both inside the growth media gel (substrate) and on the top of it. In this way, it prevented the mould from bypassing the antimould agent on the top of the gel surface or by growing their hyphae though the bulk of the gel. We envisage that this type of surface functionality (CuONPs/GLYMO/4-HPBA) can potentially be applied to a range of other inorganic nanoparticles, such as Cu_2_ONPs, Ag_2_ONPs, TiO_2_NPs, ZnONPs and others which would lead to the fabrication of superior and more environmentally friendly antimould agents that can be effective at lower nanoparticle concentrations.

## Data Availability

Data available from corresponding author upon request.

## Supplementary Materials

The following are available online at https://www.mdpi.com/2313-7673/6/1/19/s1, Figure S1: Schematics of the preparation of the Potato Glucose Agar solution for culturing mould; Figure S2: Particle size and zeta potential distribution of CuONPs; Figure S3: Zeta potential of bare CuONPs versus pH; Figure S4: Zeta potential and hydrodynamic diameter of the bare and CuONPs surfacte functionalized with GLYMO and 4- HPBA at pH 6; Figure S5: Zeta potential of *Aspergillus niger*; Figure S6: Zeta potential of *Penicillium chrysogenum* spores.
