# Supplementary material for: Enhanced Antimould Action of Surface Modified Copper Oxide Nanoparticles with Phenylboronic Acid Surface Functionality
Source: Biomimetics (Basel). 2021 Mar 15;6(1):19. doi: 10.3390/biomimetics6010019 (PMC8006150; doi:10.3390/biomimetics6010019)
Supplement: Supplementary file 1 [file biomimetics-06-00019-s001.pdf]

Supporting Information

Enhanced Antimould Action of Surface Modified Copper Oxide Nanoparticles with Phenylboronic Acid Surface Functionality

Patricia Henry [1], Ahmed F. Halbus [1,2], Zahraa H. Athab [1,3] and Vesselin N. Paunov [1,4,*]

[1] Department of Chemistry and Biochemistry, University of Hull, Hull, HU67RX, UK;
[2] Department of Chemistry, College of Science, University of Babylon, Hilla, IRAQ;
[3] Environmental Research Center, University of Babylon, Hilla, IRAQ;
[4] Department of Chemistry, Nazarbayev University, Nursultan, 010000, Kazakhstan.

* Author for correspondence: Email: vesselin.paunov@nu.edu.kz

(Biomimetics 2021)

Contents

1. Schematics of the preparation of the Potato Glucose Agar solution for culturing mould. 2

2. Particle size and zeta potential distribution of CuONPs. 3

3. Zeta potential of bare CuONPs versus pH. 4

4. Zeta potential and hydrodynamic diameter of the bare and CuONPs surfacte functionalized with GLYMO and 4- HPBA at pH 6. 5

5. Zeta potential of *Aspergillus niger* and *Penicillium chrysogenum spores*. 6

1. **A schematic overview is summarizing of the prepare a solution of Potato Dextrose Agar.**

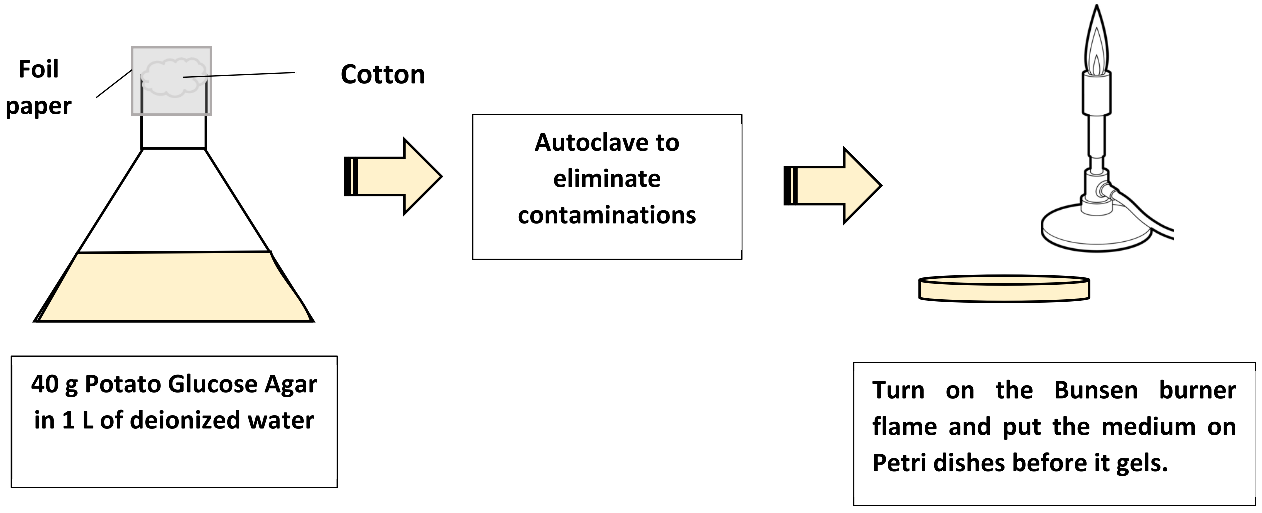

(A)

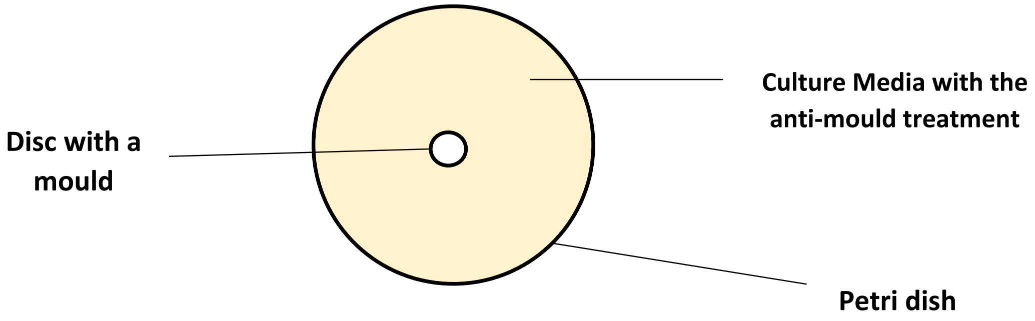

(B)

Figure S1. (A) Schematics of the preparation of the Potato Dextrose Agar (PDA) solution for culturing mould. Positioning of the disk with the mould sample in the Petri dish (Methods 1, 3 and 4 – see also Figure 1 in the main manuscript). In Method 2 the disk is impregnated with the antimould suspension, while the PDA plate is seeded with the mould.

2. Particle size and zeta potential distribution of CuONPs.

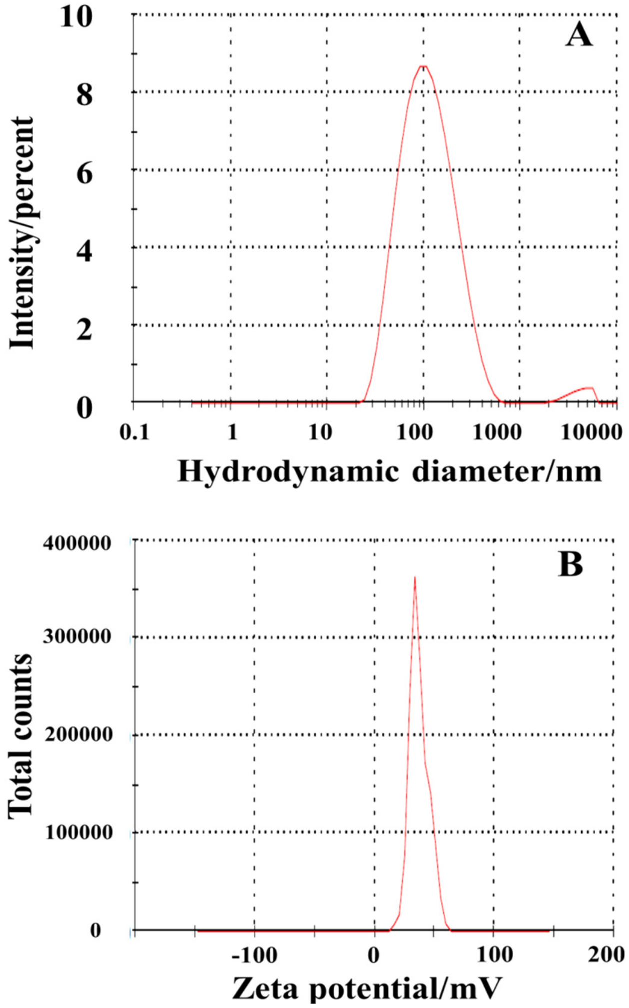

Figure S2. Plots of (A) particle hydrodynamic diameter and (B) zeta-potential distribution of CuONPs produced by annealing at 100 °C. The particle size and zeta potential of CuONPs was measured utilizing the Malvern Zetasizer Nano ZS90 at room temperature with the average data of three runs.

3. Zeta potential of bare CuONPs versus pH.

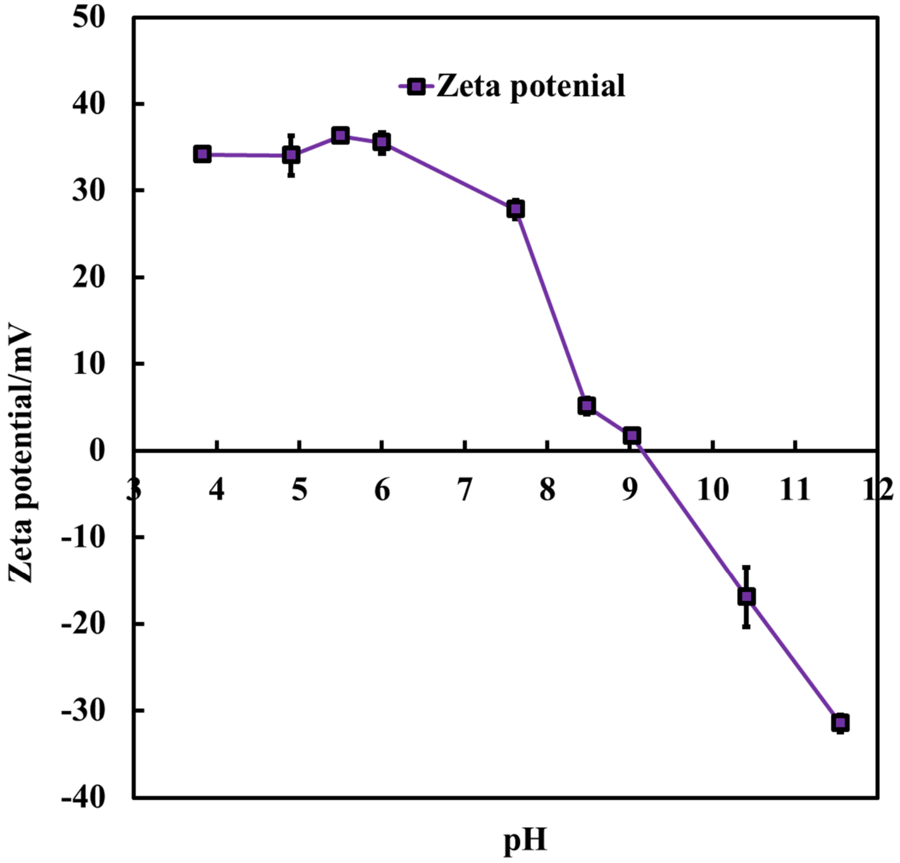

Figure S3. Zeta-potential of bare CuONPs versus pH of the aqueous suspension.

4. Zeta potential and hydrodynamic diameter of the bare and surface modified CuONPs with GLYMO and 4-HPBA at pH 6.

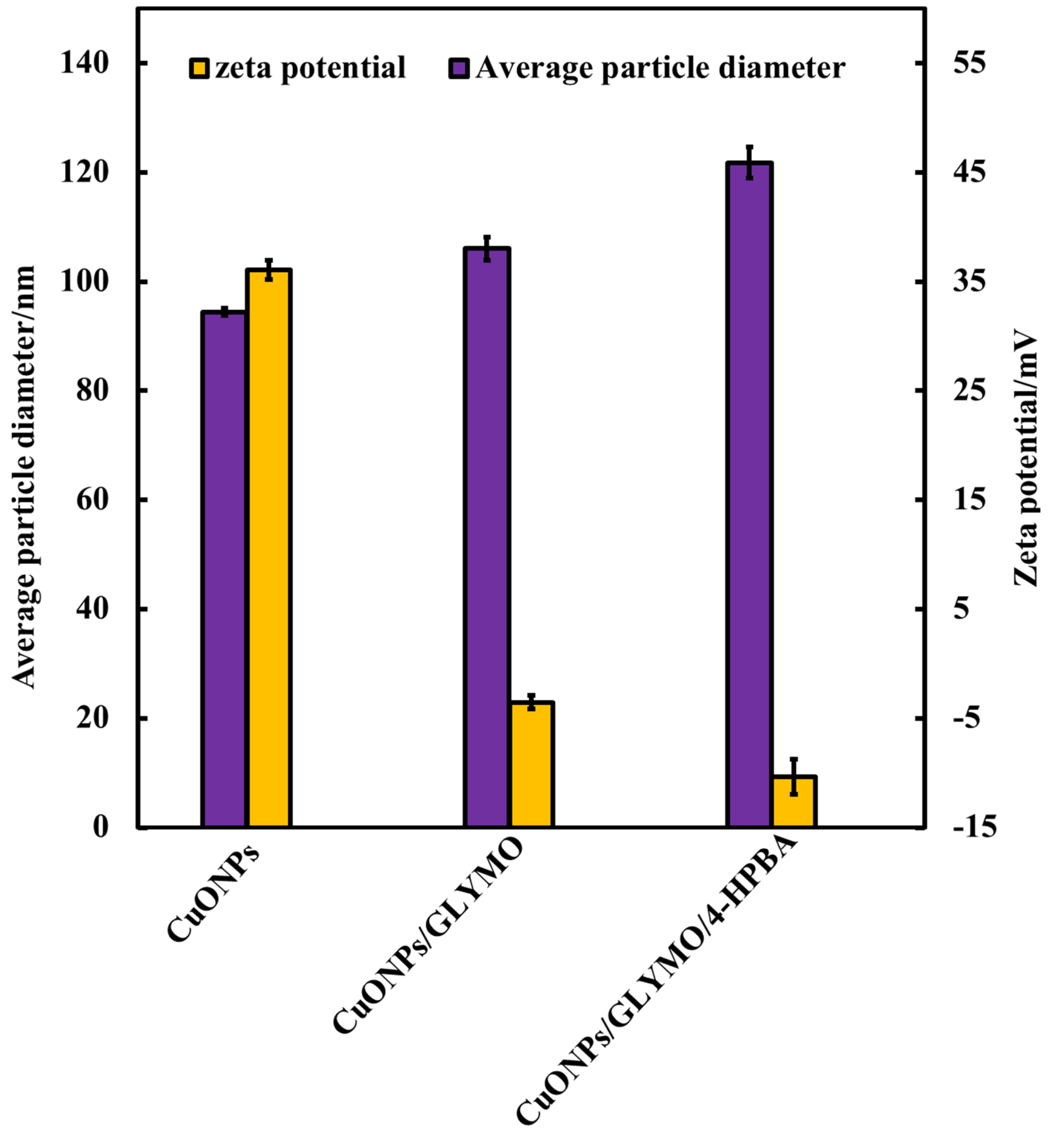

Figure S4. Zeta potential and hydrodynamic diameter of the bare and surface modified CuONPs with GLYMO and 4-HPBA, measured at room temperature at pH 6 (error bars are standard deviations).

5. Zeta potential of *Aspergillus niger* and *Penicillium chrysogenum*.

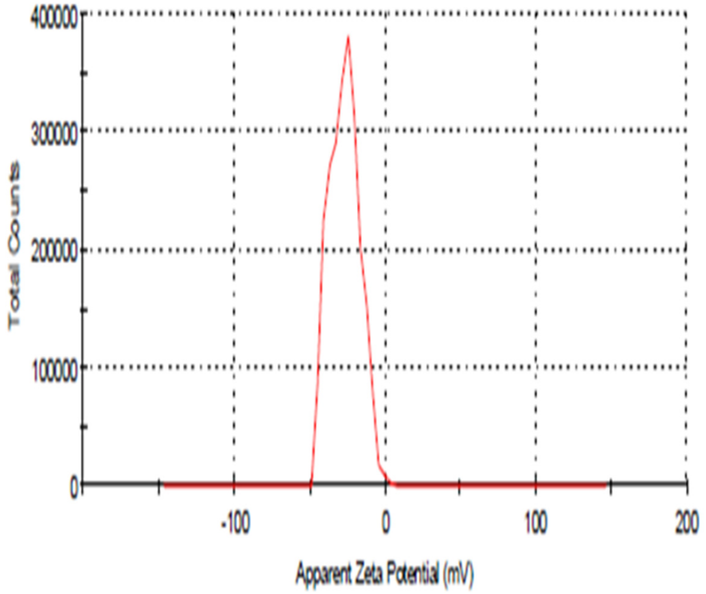

Figure S5. Zeta potential of *Aspergillus niger* spores (average ζ = -26.9 mV).

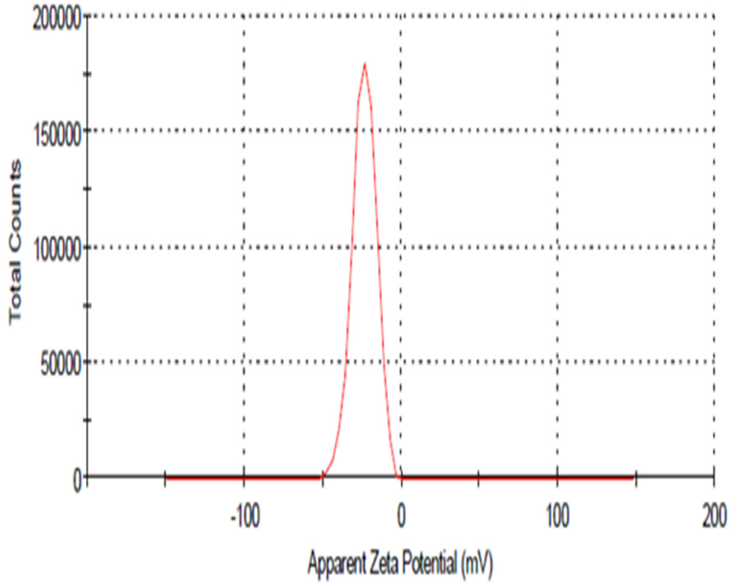

Figure S6. Zeta potential of *Penicillium chrysogenum* spores (average ζ =-23.3 mV).
